# *Plasmodium falciparum* susceptibility to anti-malarial drugs in Dakar, Senegal, in 2010: an *ex vivo* and drug resistance molecular markers study

**DOI:** 10.1186/1475-2875-12-107

**Published:** 2013-03-20

**Authors:** Bécaye Fall, Aurélie Pascual, Fatoumata D Sarr, Nathalie Wurtz, Vincent Richard, Eric Baret, Yaya Diémé, Sébastien Briolant, Raymond Bercion, Boubacar Wade, Adama Tall, Bruno Pradines

**Affiliations:** 1 Laboratoire d’étude de la chimiosensibilité du paludisme, Fédération des laboratoires, Hôpital Principal de Dakar, Dakar, Sénégal; 2 Unité de Parasitologie, Département d’Infectiologie de Terrain, Institut de Recherche Biomédicale des Armées, Marseille, France; 3 Aix Marseille Université, Unité de Recherche sur les Maladies Infectieuses et Tropicales Emergentes, UM 63, CNRS 7278, IRD 198, Marseille, Inserm 1095, France; 4 Centre National de référence du Paludisme, Marseille, France; 5 Unité d’Epidémiologie des Maladies Infectieuses, Institut Pasteur de Dakar, Dakar, Sénégal; 6 Chefferie, Hôpital Principal de Dakar, Dakar, Sénégal

**Keywords:** Malaria, *Plasmodium falciparum*, Anti-malarial, *in vitro*, Resistance, Molecular marker, Senegal

## Abstract

**Background:**

In 2006, the Senegalese National Malaria Control Programme recommended artemisinin-based combination therapy (ACT) as the first-line treatment for uncomplicated malaria. Since the introduction of ACT, there have been very few reports on the level of resistance of *P. falciparum* to anti-malarial drugs. To determine whether parasite susceptibility has been affected by the new anti-malarial policies, an *ex vivo* susceptibility and drug resistance molecular marker study was conducted on local isolates obtained from the Centre de santé Elizabeth Diouf (Médina, Dakar, Senegal).

**Methods:**

The prevalence of genetic polymorphisms in genes associated with anti-malarial drug resistance, i.e., *pfcrt*, *pfdhfr*, *pfdhps* and *pfmdr1*, were evaluated for a panel of 165 isolates collected from patients recruited from 17 August 2010 to 6 January 2011. The malaria isolates were assessed for susceptibility to chloroquine (CQ); quinine (QN); monodesethylamodiaquine (MDAQ), the active metabolite of amodiaquine; mefloquine (MQ); lumefantrine (LMF); dihydroartemisinin (DHA), the active metabolite of artemisinin derivatives; and doxycycline (DOX) using the *Plasmodium* lactate dehydrogenase (pLDH) ELISA.

**Results:**

The prevalence of the *in vitro* resistant isolates, or isolates with reduced susceptibility, was 62.1% for MQ, 24.2% for CQ, 10.3% for DOX, 11.8% MDAQ, 9.7% for QN, 2.9% for LMF and 0% for DHA. The *Pfcrt* 76T mutation was identified in 43.6% of the samples. The *pfmdr1* 86Y, 184F and 1246Y mutations were found in 16.2%, 50.0% and 1.6% of the samples, respectively. The *pfdhfr* 108N, 51I and 59R mutations were identified in 81.9%, 77.4% and 79.4% of the samples, respectively. The double mutant (108N and 51I) was detected in 75.5% of the isolates, and the triple mutant (108N, 51I and 59R) was detected in 73.6% of the isolates. The *pfdhps* 437G, 436A and 613S mutations were found in 54.4%, 38.6% and 1.2% of the samples, respectively. There was only one double mutant, 437G and 540E, and one quintuple mutant, *pfdhfr* 108N, 51I and 59R and *pfdhps* 437G and 540E. The prevalence of the quadruple mutant (*pfdhfr* 108N, 51I and 59R and *pfdhps* 437G) was 36.7%.

**Conclusions:**

The results of this study indicate that an intensive surveillance of the *in vitro P. falciparum* susceptibility to anti-malarial drugs must be conducted in Senegal.

## Background

During the past 20 years, many strains of *Plasmodium falciparum* have become resistant to chloroquine and other anti-malarial drugs [1]. One strategy for reducing malaria prevalence is the use of drugs in combination. Drug combinations help prevent the development of resistance to each component drug and reduce the overall transmission of malaria [2]. In response to increasing chloroquine resistance, Senegal switched in 2004, to sulphadoxine-pyrimethamine with amodiaquine as the first-line therapy. In 2006, the Senegalese National Malaria Control Programme recommended artemisinin-based combination therapy (ACT) as the first-line treatment for uncomplicated malaria. The combination sulphadoxine-pyrimethamine and amodiaquine treatment was changed to artemether-lumefantrine and artesunate-amodiaquine. Since 2006, more than 1.5 million ACT-based treatments have been administered in Senegal [3]. In 2006, the Senegalese National Malaria Control Programme also recommended testing for all suspected cases of malaria with the *P. falciparum* histidine-rich protein 2 (PfHRP2)-based rapid diagnostic test (RDT). Since this time, ACT use has been restricted to confirmed malaria cases to reduce drug pressure. In 2009, 184,170 doses of ACT were dispensed in Senegal [4].

Malaria is transmitted in Dakar and its surrounding suburbs with a spatial heterogeneity of the human biting rate, which ranged from 0.1 to 250 bites per person per night during the rainy season from 2007 to 2010 [5,6]. In 2008 to 2009, the human biting rate was 0.7 bites per person per night during the rainy season in Médina, a district of the south of Dakar [5]. In 2008, the *P. falciparum* prevalence varied from 0.9% to 7.4% in asymptomatic women and children in Dakar [7]. Morbidity in public health facilities decreased from 17.9% in 2007 to 2.6% in 2008 in Dakar [8].

Since the introduction of ACT, there have been very few reports on the level of resistance of *P. falciparum* to anti-malarial drugs. To determine whether parasite susceptibility has been affected by the new anti-malarial policies, an *ex vivo* susceptibility study was conducted on local isolates from Dakar obtained from the Centre de santé Elizabeth Diouf (Médina, Dakar, Senegal). The malaria isolates were assessed for susceptibility to chloroquine (CQ), quinine (QN), monodesethylamodiaquine (MDAQ), the active metabolite of amodiaquine, mefloquine (MQ), lumefantrine (LMF), dihydroartemisinin (DHA), the active metabolite of artemisinin derivatives and doxycycline (DOX).

In addition, the prevalence of genetic polymorphisms in genes associated with anti-malarial drug resistance was evaluated. The genes of interest included *P. falciparum* CQ resistance transporter (*pfcrt*) for CQ [9], *P. falciparum* dihydrofolate reductase (*pfdhfr*) for pyrimethamine [10], *P. falciparum* dihydropteroate synthase (*pfdhps*) for sulphadoxine [11] and *P. falciparum* multidrug resistance 1 (*pfmdr1*) for mefloquine resistance [12] and potentially for quinoline resistance [13,14].

The *pfcrt* gene was first identified in 2000 [9]. To date, at least 20 point mutations have been described [9,15,16], but only one is the reference mutation (K76T), which is a marker of the CQ-resistant phenotype. This mutation is often associated with other mutations in the *pfcrt* gene, whose role is not yet defined. The odds ratio (OR) for CQ failure associated with the 76T mutation was 2.1 (95% confidence interval: 1.5-3.0, meta-analysis of 13 studies) for a 14-day follow-up and 7.2 (95%CI: 4.5-11.5, meta-analysis of 12 studies) for a 28-day follow-up [17]. However, the existence of CQ-susceptible strains associated with the 76T mutation suggests that other genes could be involved in the resistance to chloroquine.

The S108N mutation in the *pfdhfr* gene is associated with resistance to anti-folate drugs [18]. The OR for sulphadoxine-pyrimethamine failure associated with S108N was 3.5 (95%CI: 1.9-6.3, meta-analysis of 10 studies) for a 28-day follow-up [17]. The additional mutations N51I, C59R or I164L increase the level of *in vitro* resistance to anti-folate drugs and sulphadoxine-pyrimethamine. The OR values for codon 51 and 59 single mutants were 1.7 (95%CI: 1.0-3.0) and 1.9 (95%CI: 1.4-2.6), respectively [17]. The triple mutation (51 + 59 + 108) increases the risk of *in vivo* resistance to sulphadoxine-pyrimethamine by 4.3 (95%CI: 3.0-6.3, meta-analysis of 22 28-day studies) [17].

Sulphones (dapsone) and sulphonamides (sulphadoxine) are inhibitors of the *P. falciparum* DHPS [19]. The mutations S436A, S436F, A437G and K540E are involved in resistance to sulphadoxine [11]. The single mutation A437G and the double mutation A437G + K540E increase the risk of *in vivo* resistance to sulphadoxine-pyrimethamine by 1.5 (95%CI: 1.0-2.4, meta-analysis of 12 studies) and 3.9 (95%CI: 2.6-5.8, meta-analysis of 10 studies), respectively [17].

The quintuple mutant of *pfdhfr* (codons 51 + 59 + 108) plus *pfdhps* (codons 437 + 540) increases the risk of *in vivo* resistance to sulphadoxine-pyrimethamine by 5.2 (95%CI: 3.2-8.8, meta-analysis of 3 studies) [17]. *Pfmdr1*, which encodes a 162 kDa protein named *P. falciparum* homologue of the P-glycoprotein (Pgh1), is located on chromosome 5. Field work has shown that the predictive value for CQ resistance and point mutations in the *pfmdr1* sequence resulting in amino acid changes varies depending on the geographic area [20,21]. Five point mutations have been described: N86Y, Y184F, S1034C, N1042D and D1246Y. Point mutations, most notably N86Y, have been associated with a decrease in the CQ susceptibility [22]. However, in some of these epidemiological studies, the number of CQ-susceptible samples is too limited to provide a statistically meaningful analysis [21,23]. Using precautions, no relationship or only weak relationships are established between CQ resistance and mutations in *pfmdr1* in *P. falciparum*[24]. However, the risk of therapeutic failure with CQ is greater for patients harbouring the 86Y mutation, with an OR of 2.2 (95%CI: 1.6-3.1) for a 14-day follow-up and 1.8 (95%CI: 1.3-2.4) for a 28-day follow-up [17]. The combination of *pfmdr1* 86Y and *pfcrt* 76T increases the risk of *in vivo* resistance to CQ by 3.9 (95%CI: 2.6-5.8, meta-analysis of 5 studies) [17].

In addition, the risk of therapeutic failure with amodiaquine is greater for patients harbouring the 86Y mutation with an OR of 5.4 (95%CI: 2.6-11.2, meta-analysis of six studies) [17]. This mutation increases the risk of failure with amodiaquine plus sulphadoxine-pyrimethamine by 7.9 [25].

It has been shown through heterologous expression that *pfmdr1* mutations at codons 1034 and 1042 abolish or reduce the level of resistance to mefloquine [26]. Moreover, transfection with a wild-type *pfmdr1* allele at codons 1034, 1042 and 1246 confers mefloquine resistance to susceptible parasites [27]. However, mutations at codons 1034, 1042 and 1246 of *Pfmdr1* in *P. falciparum* isolates are not sufficient to explain variations in mefloquine susceptibility [28]. Analyses of *P. falciparum* isolates showed an association between mutation at codon 86 and an increase in susceptibility to mefloquine, halofantrine or artemisinin derivatives [12,29,30].

## Methods

### *Plasmodium falciparum* isolates

In total, 165 patients (63 females, 3 to 70 years old and 102 males, 2 to 67 years old) with malaria were recruited from 17 August 2010 to 6 January 2011 at the Centre de santé Elizabeth Diouf. Venous blood samples were collected in Vacutainer® ACD tubes (Becton Dickinson, Rutherford, NJ, USA) prior to patient treatment and transferred to the Hôpital Principal de Dakar within six hours. Parasitaemia ranged from 0.001% to 20% in the male group and from 0.001% to 10.6% in the female group. Informed verbal consent was obtained from patients and/or their parents before blood collection. Assessment of *P. falciparum* susceptibility to anti-malarial drugs was realised with the same venous blood sample used for diagnostic purposes. The study was reviewed and approved by the ethical committee of the Pasteur Institute and the Hôpital Principal de Dakar. Patients were treated by artemether-lumefantrine or artesunate-amodiaquine (depending on availability).

Thin blood smears were stained using a RAL kit (Réactifs RAL, Paris, France) and were examined to determine the *P. falciparum* density and to confirm monoinfection. Parasitized erythrocytes were washed three times in RPMI 1640 medium (Invitrogen, Paisley, UK) buffered with 25 mM HEPES and 25 mM NaHCO_3_. If parasitaemia exceeded 0.5%, infected erythrocytes were diluted to 0.5% with uninfected erythrocytes (human blood type A+) and re-suspended in RPMI 1640 medium supplemented with 10% human serum (Abcys S.A. Paris, France), for a final haematocrit of 1.5%.

### Drugs

CQ, QN, DHA and DOX were purchased from Sigma (Saint Louis, MO, USA). MDAQ was obtained from the World Health Organization (Geneva, Switzerland), MQ was purchased from Roche (Paris, France), and LMF was purchased from Novartis Pharma (Basel, Switzerland). QN, MDAQ, MQ, DHA and DOX were first dissolved in methanol and then diluted in water to final concentrations ranging from 5 nM to 3200 nM for QN, 1.56 nM to 1000 nM for MDAQ, 3.2 nM to 400 nM for MQ, 0.1 nM to 100 nM for DHA and 0.1 μM to 502 μM for DOX. CQ was re-suspended and diluted in water to final concentrations ranging from 5 nM to 3200 nM. LMF was re-suspended and diluted in ethanol to obtain final concentrations ranging from 0.5 nM to 310 nM.

The batches of plates were tested and validated on the CQ-susceptible 3D7 strain (West-Africa) and the CQ-resistant W2 strain (Indochina) (MR4, Virginia, USA) in 3 to 6 independent experiments using the same conditions described in the paragraph below. The two strains were synchronised twice with sorbitol before use [31], and clonality was verified every 15 days using PCR genotyping of the polymorphic genetic markers *msp1* and *msp2* and microsatellite loci [32,33] and each year by an independent laboratory from the Worldwide Anti-malarial Resistance Network (WWARN).

#### *Ex vivo* assay

For the *in vitro* isotopic microtests, 200 μl of synchronous parasitized red blood cells (final parasitaemia, 0.5%; final haematocrit, 1.5%) was aliquoted into 96-well plates pre-dosed with anti-malarial drugs. The plates were incubated in a sealed bag for 42 h at 37°C with the atmospheric generators for capnophilic bacteria, Genbag CO2® at 5% CO_2_ and 15% O_2_ (BioMérieux, Marcy l’Etoile, France) [34]. After thawing the plates, haemolysed cultures were homogenised by vortexing the plates. Both the success of the drug susceptibility assay and the appropriate volume of haemolysed culture to use for each assay were determined for each clinical isolate during a preliminary pLDH ELISA. This pre-test and the subsequent ELISAs were performed using a commercial kit (ELISA-Malaria antigen test, ref 750101, DiaMed AG, Cressier, Morat, Switzerland) as previously described [35]. The optical density (OD) of each sample was measured with a spectrophotometer (Multiskan EX, Thermo Scientific, Vantaa, Finland).

The concentration at which the drugs were able to inhibit 50% of parasite growth (IC_50_) was calculated with the inhibitory sigmoid E_max_ model, with estimation of the IC_50_ through non-linear regression using a standard function of the R software (ICEstimator version 1.2) [36]. IC_50_ values were validated only if the OD ratio (OD at concentration 0 / OD at concentration max) was greater than 1.8 and the confidence interval ratio (upper 95% confidence interval of the IC_50_ estimation/lower 95% confidence interval of the IC_50_ estimation) was less than 2.0 [36].

### Nucleic acid extraction

The total genomic DNA of each strain was isolated using the QIAamp® DNA Mini kit according to the manufacturer’s recommendations (Qiagen, Germany).

### *Pfcrt* single-nucleotide polymorphisms (SNPs)

A 546-nucleotide fragment of the *pfcrt* gene (containing codon 76) was amplified by PCR using CRTP1-sense 5^′^-CCG TTA ATA ATA AAT ACA CGC AG-3^′^ and CRTP1-antisense 5^′^-CGG ATG TTA CAA AAC TAT AGT TAC C-3^′^ primers [37]. The reaction mixture for PCR amplifications included 2.5 μl of genomic DNA, 2.5 μl of 10X reaction buffer (Eurogentec), 0.5 μM of each primer, 200 μM of a deoxynucleoside triphosphate mixture (dGTP, dATP, dTTP and dCTP) (Euromedex, Souffelweyersheim, France), 2.5 mM MgCl_2_ and 1 unit of RedGoldStar® DNA polymerase (Eurogentec) in a final volume of 25 μl. The thermal cycler (T3 Biometra, Archamps, France) was programmed as follows: an initial 94°C incubation for 5 min; 40 cycles of 94°C for 20 sec, 56°C for 20 sec and 60°C for 40 sec; and a final 5-min extension step at 60°C. The PCR products were loaded on a 1.5% agarose gel containing 0.5 μg/mL ethidium bromide. The PCR products were diluted 1:100 in distilled water, and 2.5 μl of the final dilution was used for the second PCR. This PCR amplified a 275 bp segment around the mutation using a common inner primer CRTP3-sense 5^′^-TGA CGA GCG TTA TAG AG-3^′^ coupled with either CRTP4m-antisense 5^′^-GTT CTT TTA GCA AAA ATT G-3^′^ (detects the 76T codon), or CRTP4w-antisense 5^′^-GTT CTT TTA GCA AAA ATT T-3^′^ (detects the 76K codon) [15]. The reaction mixture for the PCR amplifications included 2.5 μl of diluted PCR product, 2.5 μl of 10X reaction buffer (Eurogentec), 0.5 μM of each primer, 200 μM deoxynucleoside triphosphate mixture, 1.5 mM MgCl_2_ and 0.75 U of RedGoldStar® DNA polymerase (Eurogentec) in a final volume of 25 μl.

The PCR conditions were as follows: initiation at 94°C for 5 min; 15 cycles at 94°C for 20 sec, 48.5°C for 20 sec and 64°C for 40 sec; and a final 5 min extension step at 64°C. Purified genomic DNA from *P. falciparum* clones 3D7 (chloroquine sensitive) and W2 (chloroquine resistant) were used as positive controls, and water and human DNA were used as negative controls. The PCR products from the amplification reactions were evaluated by electrophoresis on 2% agarose gels.

### *Pfmdr1* SNPs

Two primer pairs were used to amplify *pfmdr1* fragments carrying the five key codons [38]. A 590-base pair fragment was amplified with the primer pair sense 5^′^-AGA GAA AAA AGA TGG TAA CCT CAG-3^′^ and antisense 5^′^-ACC ACA AAC ATA AAT TAA CGG-3^′^ to determine the sequences of codons 86 and 184 (MDR1-1), and a second fragment (968 base pairs) was amplified with the primer pair sense 5^′^-CAG GAA GCA TTT TAT AAT ATG CAT-3^′^ and antisense 5^′^-CGT TTA ACA TCT TCC AAT GTT GCA-3^′^ to determine the sequences of codons 1034, 1042, and 1246 (MDR1-2) [38]. The reaction mixture consisted of approximately 2.5 μl of genomic DNA, 0.5 μM of forward and reverse primers, 2.5 μl of 10X reaction buffer (Eurogentec), 2.5 mM MgCl_2_, 200 μM deoxynucleoside triphosphate mixture (dGTP, dATP, dTTP and dCTP) (Euromedex, Souffelweyersheim, France) and 1 U of RedGoldStar® DNA polymerase (Eurogentec) in a final volume of 25 μl. The thermal cycler (T3 Biometra) was programmed for MDR1-1 as follows: an initial step at 94°C for 5 min; 40 cycles of 94°C for 30 sec, 52°C for 30 sec and 72°C for 1 min; and a final 10-min extension step at 72°C. For MDR1-2 the parameters were as follows: an initial step at 94°C for 5 min; 40 cycles of 94°C for 30 sec, 56°C for 1 min and 72°C for 1 min 30 sec; and a final 10-min extension step at 72°C. The PCR products were loaded on a 1.5% agarose gel containing 0.5 μg/mL ethidium bromide. Amplicons were purified using the QIAquick 96 PCR BioRobot Kit and an automated protocol on the BioRobot 8000 workstation (Qiagen, Courtaboeuf, France). The purified fragments were sequenced using the BigDye Terminator v3.1 Cycle Sequencing Kit (Applied Biosystems) using the primers described above. The sequencing reaction products were purified using the BigDye XTerminator® Purification Kit (Applied Biosystems) in accordance with the manufacturer’s instructions. The purified products were sequenced using an ABI Prism 3100 analyser (Applied Biosystems). The sequences were analysed using Vector NTI advance (TM) software (version 11, Invitrogen, Cergy Pontoise, France).

### *Pfdhfr* SNP

A 562-bp fragment corresponding to the coding region of *pfdhfr* was amplified using the following primers: sense 5^′^-ACG TTT TCG ATA TTT ATG C-3^′^ and antisense 5^′^ TCA CAT TCA TAT GTA CTA TTT ATT C-3^′^[39]. The reaction mixture contained 2.5 μl of genomic DNA, 2.5 μl of 10X reaction buffer (Eurogentec), 0.5 μM each primer, 2.5 mM MgCl_2_, 200 μM deoxynucleoside triphosphate mixture (dGTP, dATP, dTTP and dCTP) (Euromedex, Souffelweyersheim, France) and 1 U of RedGoldStar® DNA polymerase (Eurogentec) in a final volume of 25 μl. The PCR conditions were as described in [39]. The amplified fragments were purified, sequenced (with the primers used for PCR) and analysed as described above.

### *Pfdhps* SNP

A 672-bp fragment corresponding to the coding region of *pfdhps* was amplified using the following primers: sense 5^′^-GTT GAA CCT AAA CGT GCT GT-3^′^ and antisense 5^′^-TTC ATC ATG TAA TTT TTG TTG TG-3^′^[39]. The fragment was amplified as described for *pfdhfr*, and the PCR conditions were as described in [39]. The amplified fragments were purified, sequenced (with the primers used for PCR) and analysed as described above.

### Data and statistical analysis

IC_50_ values were analysed after logarithmic transformation and expressed as the geometric mean of the IC_50_ and a confidence interval of 95% (CI95%). Using the *Plasmodium* lactate dehydrogenase (pLDH) ELISA under Genbag conditions, the cut-off values for *in vitro* resistance, or reduced susceptibility, were 77 nM, 61 nM, 115 nM, 12 nM, 611 nM, 30 nM and 37 μM for CQ, MDAQ, LMF, DHA, QN, MQ and DOX, respectively [40].

## Results

Out of the 165 patients recruited at the Centre de santé Elizabeth Diouf, 63 were tested *ex vivo*, and 34 isolates were successfully cultured. The average parameter estimates for the seven anti-malarial drugs utilised against the *P. falciparum* isolates are given in Table 1. The prevalence of *P. falciparum* isolates with decreased susceptibility to MQ *in vitro* reached 62.1%.

**Table 1:** ***Ex vivo *susceptibility of 34 *Plasmodium falciparum *isolates from Dakar to chloroquine (CQ), monodesethylamodiaquine (MDAQ), lumefantrine (LMF), dihydroartemisinin (DHA), quinine (QN), mefloquine (MQ) and doxycycline (DOX)**

**Drug**	**No**	**Isolate IC**_**50**_				**Resistance or reduced susceptibility**	
		**Mean**	**CI95%**	**Min**	**Max**	**Cut-off**	**%**
CQ	34	41.3 nM	26.7-64.0	5.1	1958	77 nM	24.2
MDAQ	34	19.4 nM	13.3-28.4	1.6	179	61 nM	11.8
LMF	34	9.8 nM	6.3-15.2	0.77	139	115 nM	2.9
DHA	32	3.6 nM	2.8-4.6	1.2	11.0	12 nM	0
QN	34	141.3 nM	93.7-213.0	5.2	1195	611 nM	9.7
MQ	32	30.1 nM	22.2-41.0	5.3	86.9	30 nM	62.1
DOX	32	9.2 μM	6.5-12.9	0.5	41.9	37 μM	10.3

Mean: geometric mean.

CI95%: 95% confidence interval.

*Pfcrt* was examined in 156 of the samples. Within the *pfcrt* gene, the 76T mutation was identified in 43.6% of the samples. Eighteen samples (10.3%) were mixed, yielding both K76 and 76T.

The results for *pfmdr1* polymorphisms are shown in Table 2. The 86Y mutation was identified in 14.9% of the tested samples. A mutation in codon 184 (184F) was identified in 49.4% of the isolates. Eighteen of the 24 isolates with the 86Y mutation were also mutated at codon 184 (184F). No new SNP was detected in the *pfmdr1* gene.

**Table 2:** **Number (no) and frequency (%) of the *Pfmdr1 *mutations (codons 86, 184, 1034, 1042, and 1246)**

**Codon**	**No**	**Wild type no (%)**	**Mutated no (%)**	**Wild type/Mutated no (%)**
N86Y	161	135 (83.9)	24 (14.9)	2 (1.2)
Y184F	156	78 (50.0)	77 (49.4)	1 (0.6)
S1034C	153	153 (100)	0 (0)	0 (0)
N1042D	156	156 (100)	0 (0)	0 (0)
D1246Y	156	154 (98.7)	2 (1.3)	0 (0)

The results for *pfdhfr* polymorphisms are presented in Table 3. There was a mutation in 81.9% of the samples for codon 108 (S108N/T), in 77.4% for codon 51 (N51I) and in 79.4% for codon 59 (C59R). The double mutant (108N and 51I) was detected in 75.5% of the isolates, and the triple mutant (108N, 51I and 59R) was detected in 73.6% of samples.

**Table 3:** **Number (no) and frequency (%) of the *Pfdhfr *mutations (codons 108, 51, 59, 16, and 164)**

**Codon**	**no**	**Wild type no (%)**	**Mutated no (%)**
S108N	155	28 (18.1)	127 (81.9)
N51I	155	35 (22.6)	120 (77.4)
C59R	155	32 (20.6)	123 (79.4)
A16V	155	155 (100)	0 (0)
I164L	153	153 (100)	0 (0)

The results for the *pfdhps* polymorphisms are presented in Table 4. There was a mutation in 47.5% of the samples for codon 437 (A437G), in 33.5% for codon 436 (S436A), in 1.2% for codon 613 (A613S) and in 0.6% for codon 540 (K540E). There was only one (0.6%) double mutant (437G and 540E) and one (0.6%) quintuple mutant (*pfdhfr* 108N, 51I and 59R and *pfdhps* 437G and 540E), and 58 isolates (36.7%) were quadruple mutants (*pfdhfr* 108N, 51I and 59R and *pfdhps* 437G).

**Table 4:** **Number (no) and frequency (%) of the *Pfdhps *mutations (codons 437, 436, 540, 581, and 613)**

**Codon**	**no**	**Wild type no (%)**	**Mutated no (%)**	**Wild type/Mutated no (%)**
A437G	158	72 (45.6)	75 (47.5)	11 (6.9)
S436A	158	97 (61.4)	53 (33.5)	8 (5.1)
K540E	160	159 (99.4)	1 (0.6)	0 (0)
A581G	160	160 (100)	0 (0)	0 (0)
A613S	160	158 (98.8)	2 (1.2)	0 (0)

## Discussion

This report describes the evaluation of the *ex vivo* susceptibility of *P. falciparum* isolates, taken from patients in Dakar, to seven standard anti-malarial drugs and the prevalence of several molecular markers involved in anti-malarial drug resistance. The majority of the patients, recruited at the Centre de santé Elizabeth Diouf (Médina, Dakar) from August 2010 to January 2011, said that they did not leave Dakar and its surrounding suburbs during the month preceding their malaria attack.

The prevalence of isolates with reduced susceptibility to MQ remains high (62.1%) in Dakar, but relatively stable compared with the previous year (55%) [40]. The level of *in vitro* resistance to MQ has increased since previous studies conducted in Senegal. In Dakar, the percent of isolates with decreased susceptibility was 17% in 2001 [41] and 13% in 2002 [33]. In Dielmo and Ndiop (280 km south-east of Dakar), the prevalence of *in vitro* resistance to MQ was 22% in 1995 [42] and 15% in 1999 [43,44]. Prophylaxis failure with MQ has been previously described in Senegal [45], and MQ is one of the three anti-malarial drugs recommended for travellers as an anti-malarial prophylaxis in Senegal. Clinical trials are in progress to evaluate the efficacy of MQ for intermittent preventive treatment of infants and pregnant women, while MQ is still used for the treatment of uncomplicated malaria in infants in Dakar. Nevertheless, MQ has been employed relatively infrequently in Africa compared to Asia. The combination of artesunate-mefloquine, which is administered to patients in Asia, is not yet used in Senegal. However, scientific data are not available for MQ monotherapy, and very few data are available on the *in vitro* decreased susceptibility to MQ and its clinical implications in Africa. It is important to monitor the evolution of *P. falciparum* susceptibility to MQ, to archive suspicious isolates and to correlate clinical outcomes with pharmacokinetic and phenotypic responses and with molecular markers.

As far back as 1988, *in vitro P. falciparum* resistance to CQ was reported in Dakar, and reports of resistance in other regions of the country followed shortly [46]. From 1991 to 1995, parasitological failures were observed in 21% of patients in Pikine and in 23% of patients in another region of Senegal [47]. The *in vitro* resistance to CQ increased from 1995 to 1999 in Dielmo with 32% resistance in 1995 [48] compared to 49% resistance in 1996 [49], 44% resistance in 1997 [50] and 55% resistance in 1999 [43]. In 2010, the prevalence of *in vitro* resistance to CQ in Dakar was low and stable in comparison with the previous year (24.2% versus 22%) [40]. These data are consistent with previous work on *in vitro* resistance in Thies in 2007 (23% of isolates exhibiting CQ resistance) [51]. The evolution of susceptibility to CQ is confirmed by evaluation of molecular markers of CQ resistance, and mutations in *pfcrt* have been shown to be correlated with CQ resistance in different parts of the world [52]. The prevalence of the *pfcrt* 76T mutation has decreased since 2004 in Dakar. From 2000 to 2001 in Guediawaye, a suburb of Dakar, a prevalence of 92% of was observed for the 76T mutation in pregnant women with malaria [53]. In Pikine, another suburb of Dakar, the prevalence of the 76T mutant was 79% in 2000 [54], 63.9% in 2001 [55] and 59.5% in 2004 [56]. In 2002, the prevalence of the *pfcrt* 76T mutation was 65% in patients hospitalised for malaria at the Hôpital Principal de Dakar [33]. From 2001 to 2002, the prevalence of the *pfcrt* 76T mutation was 75.8% in pregnant women taking chloroquine prophylaxis in Thiadiaye (84 km southeast of Dakar) [57]. In this study, the *pfcrt* 76T mutation was identified in 43.6% of the patients recruited from August 2010 to January 2011. These data are consistent with previous works on CQ molecular resistance in Dakar in 2009 (37.8%) [58], in central Senegal (Mbour, Fatick and Bambey) in 2009 (29.3%) and in 2010 (25.1%); and in south Senegal (Tambacounda, Velingara and Saraya) with 2010 (34.5%) and in 2011 (28.8%) [59]. However, in Pikine, the prevalence of the *pfcrt* 76T mutation ranged from 64% to 79% before CQ withdrawal (2000 to 2003) [54,55,60], the prevalence then decreased to 47-60% [56,60] while amodiaquine plus pyrimethamine-sulphadoxine was the first-line treatment (2004–2005); this prevalence has increased slightly to 59% since ACT has been implemented (2006 to 2009) [60].

This decrease in CQ resistance parallels the withdrawal of CQ treatment and the introduction of ACT in 2002 in Senegal. However, in 2003, CQ was still being administered to patients. The prevalence of CQ in the urine ranged from 14.5% to 47.5% in two- to nine-year-old children from northern Senegal and from 9.0% to 21.4% in children from southern Senegal [61]. In 2006, the Senegalese National Malaria Control Programme recommended ACT as the first-line treatment for uncomplicated malaria, in this same year, Senegal reported 10.6% chloroquine use and 9.7% ACT use [62]. In Dakar in 2006, CQ represented 5.1% of the anti-malarial drugs used in children [63] and 3.5% in 2009 [64]. Since 2006, more than 1.5 million ACT treatments have been administered in Senegal [3], and 184,170 doses of ACT were dispensed in 2009 [4]. A reduction in CQ resistance was also reported in Malawi after the withdrawal of CQ treatment [65]. This observation prompted an *in vivo* CQ study in Malawi five years later, in which CQ was found to be 99% effective [66]. The rapid dissemination of CQ resistance in Dielmo, despite strictly controlled anti-malarial drug use, argues against the re-introduction of CQ, at least in mono-therapy, in places where the resistant allele has dropped to very low levels following the discontinuation of CQ treatment [67]. Despite the regain of CQ susceptibility, any reintroduction would likely result in a rapid re-emergence of resistant strains.

The prevalence of isolates with *in vitro* reduced susceptibility to MDAQ slightly increased in 2010, 11.8% versus 6% in 2009 [40]. The prevalence of reduced susceptibility to MDAQ was 0% in 1996 and 1999 in Dielmo [44,48], while it was 5% in Mlomp (Casamance, south-western Senegal) in 2004 [68].

The prevalence of the *pfmdr1* mutations 86Y, 184F and 1246Y were 16.2%, 50.0% and 1.6%, respectively. In 2000 and 2001, prevalence rates of 31% and 30.6%, respectively, were observed for *pfmdr1* 86Y in Pikine [54,55], and a prevalence rate of 17.2% was observed in Dakar in 2009 [58]. However, the role of polymorphism in *pfmdr1* is still debated. The *pfmdr1* 86Y mutation has been shown to be associated with *in vivo* resistance to amodiaquine in recrudescence after monotherapy with amodiaquine [69] or after combination therapy with artesunate-amodiaquine [70]. The *pfmdr1* 1246Y mutation has also been found to be associated with *in vitro* resistance to amodiaquine [71] and with recrudescent infection after treatment with amodiaquine or amodiaquine-artesunate [70,72]. In a meta-analysis, the *pfmdr1* 86Y mutation was found to be associated with amodiaquine failure, with an odds ratio of 5.4 [17]. Based on this hypothesis, the 16.2% prevalence of *pfmdr1* of 86Y predicts that 16.2% of isolates would be resistant to amodiaquine in 2010 in Senegal. The resistance to amodiaquine has remained low even after the introduction of artesunate-amodiaquine in 2006 in Senegal. A study in Dakar and Mlomp from 1996 to 1998 showed that monotherapy with amodiaquine remained effective for treating uncomplicated malaria in areas where CQ resistance was prevalent [73]. The artesunate-amodiaquine–associated cure rates were > 99.3% in Mlomp and Keur-Socé when administered either as a single daily dose or as two daily doses [74]. The fixed-dose combination of artesunate-amodiaquine (ASAQ) exhibits a cure rate > 98.5% [75]. However, ACT efficacy and resistance must be monitored because clinical failures, or at least extended parasite clearance times, have been described in Cambodia [76,77]. In this context, it is important to implement *in vitro* and *in vivo* surveillance programmes, such as those championed by the Worldwide Antimalarial Resistance Network [78,79].

No isolate exhibited reduced *in vitro* susceptibility to DHA. This result is consistent with previous studies that did not show any parasites resistant to artesunate [33,43,44]. However, Agnamey *et al.* reported that 3% - 23% of isolates had IC_50_ values greater than 15 nM in Mlomp between 2000 and 2004 [73]. High IC_50_ values can also be found for artemisinin, with an IC_50_ > 30 nM in Dakar [51] and artesunate with an IC_50_ > 45 nM [41].

The other ACT first-line treatment for uncomplicated *P. falciparum* malaria in Senegal is the combination of artemether-lumefantrine. Only 2.9% of the isolates presented reduced susceptibility to LMF, and this prevalence did not rise in Senegal after the introduction of ACT. In 1996, 6% of isolates from Dielmo were resistant to LMF *in vitro*[80]; and 1% of the isolates were resistant to LMF in 2009 in Dakar [40].

In 2009, 7% of isolates showed low reduced susceptibility to QN, which is in accordance with previous studies in Dakar [33,40,41] and Dielmo [42-44]. Isolates with a high IC_50_ to QN were already identified in 1984 [81]. Even in areas where QN efficacy remains good, such as sub-Saharan Africa, the susceptibility of individual *P. falciparum* isolates to QN has varied widely. The IC_50_s for isolates collected in Senegal were 31 to 765 nM in 1984 (Thies and Kaolack) [81], 5 to 932 nM in 1996 (Dielmo) [49] and 6 to 1291 nM in 2009 (Dakar) [40]. The wide range in QN susceptibility and recent evidence for QN treatment failure seen across Africa [82,83] or in Senegal in a patient who spent two months in Dielmo in 2007 [84] suggest that the evolution of parasites with reduced susceptibility may contribute to QN decreased efficacy. QN used in combination with DOX in the treatment of severe malaria in Dakar.

A prevalence of 10.3% of isolates with *in vitro* reduced susceptibility to DOX was observed in Dakar in 2010, which is similar to the prevalence observed in 2009 (12%) [40]. The mean IC_50_ of DOX was similar to those estimated in Dielmo in 1998 [85,86]. The slow activity of DOX *in vitro* has a delayed effect upon growth and requires the prolonged incubation of parasites [85,87]. However, the standard 42 h test is still used to monitor DOX *in vitro* susceptibility.

The *pfdhfr* 108N mutation has been shown to be correlated with *in vitro* and *in vivo* resistance to pyrimethamine [10,17]. The OR for sulphadoxine-pyrimethamine failure associated with Ser108Asn has been shown to be 3.5 (95%CI: 1.9-6.3, meta-analysis of 10 studies) for a 28-day follow-up [17]. The additional mutations N51I, C59R or I164L increase the level of *in vitro* resistance to anti-folate drugs and sulphadoxine-pyrimethamine. The OR values for single mutants of codons 51 and 59 are 1.7 (95%CI: 1.0-3.0) and 1.9 (95%CI: 1.4-2.6), respectively [17]. In 2010, the prevalence of *pfdhfr* 108N was 81.9% in patients with malaria who were treated at the Centre de santé Elizabeth Diouf, which is similar to the results of those treated in 2009 at the Hôpital Principal de Dakar (82.4%) [58]. The triple mutation (51 + 59 + 108) increases the risk of *in vivo* resistance to sulphadoxine-pyrimethamine by 4.3 (95%CI: 3.0-6.3, meta-analysis of 22 28-day studies). Isolates carrying a combination of three mutations (108N, 51I and 59R) were associated with high-level pyrimethamine resistance and represented 73.6% of isolates. In 2002, in Dakar, the prevalence of *pfdhfr* 108N was 65%, and triple mutants were identified in 50% of the isolates [33]. In 2003, the prevalence of mutations in *pfdhfr* codon 108 was 78% in Pikine, and the prevalence of the triple mutant was 61% [88]. Additionally, in 2007, in the rural area Keur Soce, triple mutants were identified in 67% of patients treated with sulphadoxine-pyrimethamine combined with amodiaquine [89].

The *pfdhps* 437G mutation has been shown to be correlated with *in vitro* and *in vivo* resistance to sulphadoxine [11,17]. The single mutation Ala437Gly and the double mutation Ala437Gly + Lys540Glu increase the risk of *in vivo* resistance to sulphadoxine-pyrimethamine by 1.5 (95%CI: 1.0-2.4, meta-analysis of 12 studies) and 3.9 (95%CI: 2.6-5.8, meta-analysis of 10 studies), respectively [17]. In 2010, the prevalence of the *pfdhps* 437G mutation was 54.4% in patients with malaria in Dakar. Only one isolate (0.6%) carried the double mutation (437G and 540E) that is associated with high-level sulphadoxine resistance. The mutation of codon 613 (A613S) (1.2%) is very rare in Africa. In 2002, in Dakar, only 20% of isolates harboured the *pfdhps* 437G mutation [33]. In 2003, the mutation rate in *pfdhps* codon 437G was 40% in Pikine [88]. Several studies from 2006 to 2008 in Senegal showed that the prevalence of *pfdhps* 437G significantly increased after intermittent preventive treatment of infants with sulphadoxine-pyrimethamine [89,90]. Given the prevalence of the triple and quadruple mutants in the population of Dakar (73.6% for the *pfdhfr* 108N, 51I and 59R triple mutant and 36.7% for the quadruple mutant *pfdhfr* 108N, 51I and 59R and *pfdhps* 437G), the use of sulphadoxine-pyrimethamine as an intermittent preventive treatment (IPT) must be monitored. Encouragingly, only one quintuple mutant, *Pfdhfr* 108N, 51I and 59R and *Pfdhps* 437G and 540E, which is associated with high-level sulphadoxine-pyrimethamine resistance has been identified to date.

IPT with anti-malarial drugs given to all children and pregnant women once per month during the transmission season can provide a high degree of protection against malaria. Seasonal IPT with sulphadoxine-pyrimethamine and one dose of artesunate resulted in a 90% reduction in the incidence of clinical malaria in Senegal [6]. The combination of sulphadoxine-pyrimethamine and amodiaquine was more effective than the combination of sulphadoxine-pyrimethamine and artesunate or the combination of amodiaquine and artesunate in preventing malaria [91]. During IPT with sulphadoxine-pyrimethamine and piperaquine, only 3.4% of the treated children developed malaria [89]. However, the single use of sulphadoxine-pyrimethamine as seasonal IPT is inadvisable; for instance, sulphadoxine-pyrimethamine must be used in combination with amodiaquine, artesunate or piperaquine [89,92].

The introduction of ACT in 2002 in Senegal did not induce a decrease in *P. falciparum* susceptibility to individual drug components, such as DHA, MDAQ and LMF. However, the prevalence of *P. falciparum* isolates with reduced drug susceptibility to MQ increased, and clinical failures with QN have been reported in Senegal. Additionally, in Senegal, isolates with high IC_50_ values for artemisinin derivatives. Since 2004, the prevalence of chloroquine resistance has decreased, but the data argue against the re-introduction of chloroquine for mono-therapy in places where the resistant allele has dropped to very low levels following discontinuation of chloroquine treatment. The prevalence of isolates resistant to pyrimethamine is high (81.9%), with 73.6% of parasites exhibiting high-level pyrimethamine resistance. The prevalence of isolates resistant to sulphadoxine was 54.4%. Susceptibility to anti-malarial drugs remains stable between 2009 and 2010 in Dakar. Nevertheless, an intensive surveillance of the susceptibility of *P. falciparum* to anti-malarial drugs *in vitro* must be conducted in Senegal. In addition, maximising the efficacy and longevity of ACT as a tool to control malaria will critically depend on pursuing intensive research into identifying *in vitro* markers as well as implementing *ex vivo* and *in vivo* surveillance programmes. In this context, there is a need to identify molecular markers that predict ACT resistance, which can provide an active surveillance method to monitor temporal trends in parasite susceptibility.

## Competing interests

The authors declare that they have no competing interests.

## Authors’ contributions

BF, EB, YD, RB and BP carried out the *in vitro* testing of drug susceptibility. AP and NW carried out the molecular genetic studies. FDS, VR and AT supervised, carried out and coordinated the field collection of isolates from patients. RB, BW and BP conceived and coordinated the study. BP and SB analysed the data. BF, AP, NW, SB and BP drafted the manuscript. All authors read and approved the final manuscript.

## References

[B1] Le Bras J Musset L Clain J Antimalarial drug resistance Med Mal Infect 2006 36 401 405 10.1016/j.medmal.2006.05.005 16854546

[B2] White NJ Preventing antimalarial drug resistance through combinations Drug. Resist Updat 2001 1 3 9 17092790 10.1016/s1368-7646(98)80208-2

[B3] Ndiaye JLA Faye B Gueye A Tine R Ndiaye D Tchania C Ndiaye I Barry A Cisse B Lameyre V Gaye O Repeated treatment of recurrent uncomplicated *Plasmodium falciparum* malaria in Senegal with fixed-dose artesunate plus amodiaquine versus fixed-dose artemether plus lumefantrine: a randomized, open-label trial Malar J 2011 10 237 10.1186/1475-2875-10-237 21838909 PMC3171378

[B4] Thiam S Thior M Faye B Diouf ML Diouf MB Diallo I Fall FB Ndiaye JL Albertini A Lee E Jorgensen P Gaye O Bell D Major reduction in anti-malarial drug consumption in Senegal after nation-wide introduction of malaria rapid diagnostic tests Plos One 2011 6 18419 10.1371/journal.pone.0018419 PMC3071817 21494674

[B5] Gadiaga L Machault V Pagès F Gaye A Jarjaval F Godefroy L Cissé B Lacaux JP Sokhna C Trape JF Rogier C Conditions of malaria transmission in Dakar from 2007 to 2010 Malar J 2011 10 312 10.1186/1475-2875-10-312 22018223 PMC3216462

[B6] Drame PM Machault V Diallo A Cornélie S Poinsignon A Lalou R Sembène M Dos Santos S Rogier C Pagès F Le Hesran JY Remoué F IgG responses to the gSG6-P1 salivary peptide for evaluating human exposure to Anopheles bites in urban areas of Dakar Senegal. Malar J 2012 11 72 10.1186/1475-2875-11-72 PMC3337805 22424570

[B7] Diallo A Ndam NT Moussiliou A Dos Santos S Ndonky A Borderon M Oliveau S Lalou R Le Hesran JY Asymptomatic carriage of Plasmodium in urban Dakar: the risk of malaria should not be underestimated Plos One 2012 7 31300 10.1371/journal.pone.0031300 PMC3283586 22363558

[B8] Ministère de la Santé et de la Prévention Rapport sur la morbidité et la mortalité palustre au Sénégal en 2008 2010 Dakar: Programme National de Lutte contre le Paludisme http://www.pnlp.sn/UserFiles/File/donnee.pdf

[B9] Fidock DA Nomura T Talley AK Cooper RA Dzekunov SM Ferdig MT Ursos LMB Sidhu ABS Naudé B Deitsch KW Su XZ Wootton JC Roepe PD Wellems TE Mutations in the *P. falciparum* digestive vacuole transmembrane protein PfCRT and evidence for their role in chloroquine resistance Mol Cell 2000 6 861 871 10.1016/S1097-2765(05)00077-8 11090624 PMC2944663

[B10] Zolg JW Plitt JR Chen GX Palmer S Point mutations in the dihydrofolate reductase-thymidylate synthase gene as the molecular basis for pyrimethamine resistance in *Plasmodium falciparum* Mol Biochem Parasitol 1989 36 253 262 10.1016/0166-6851(89)90173-4 2677719

[B11] Wang P Read M Sims PF Hyde JE Sulfadoxine resistance in the human malaria parasite *Plasmodium falciparum* is determined by mutations in dihydropteroate synthetase and an additional factor associated with folate utilisation Mol Microbiol 1997 23 979 986 10.1046/j.1365-2958.1997.2821646.x 9076734

[B12] Price RN Cassar C Brockman A Duraisingh M van Vugt M White NJ Nosten F Krishna S The *pfmdr1* gene is associated with a multidrug-resistant phenotype in *Plasmodium falciparum* from the Western border of Thailand Antimicrob Agents Chemother 1999 43 2943 2949 10582887 10.1128/aac.43.12.2943 PMC89592

[B13] Henry M Alibert S Orlandi-Pradines E Bogreau H Fusai T Rogier C Barbe J Pradines B Chloroquine resistance reversal agents as promising antimalarial drugs Curr Drug Targets 2006 7 935 948 10.2174/138945006778019372 16918322

[B14] Henry M Alibert S Rogier C Barbe J Pradines B Inhibition of efflux of quinolines as new therapeutic strategy in malaria Curr Top Med Chem 2008 8 563 578 10.2174/156802608783955593 18473883

[B15] Nagesha HS Casey GJ Rieckmann H Fryauff DJ Laksana BS Reeder JC Maguire JD Baird J New haplotypes of the *Plasmodium falciparum* chloroquine resistance transporter (*pfcrt*) gene among chloroquine-resistant parasite isolates Am J Trop Med Hyg 2003 68 398 402 12875286

[B16] Johnson DJ Fidock DA Mungthin M Lakshmanan V Sidhu AB Bray PG Ward SA Evidence for a central role for PfCRT in conferring *Plasmodium falciparum* resistance to diverse antimalarial agents Mol Cell 2004 15 867 877 10.1016/j.molcel.2004.09.012 15383277 PMC2943419

[B17] Picot S Olliaro P de Monbrison F Bienvenu AL Price RN Ringwald P A systematic review and meta-analysis of evidence for correlation between molecular markers of parasite resistance and treatment outcome in *falciparum* malaria Malar J 2009 8 89 10.1186/1475-2875-8-89 19413906 PMC2681474

[B18] Sibley CH Hyde JE Sims PF Plowe CV Kublin JG Mbenu EK Cowman AF Winstanley PA Watkins WM Nzila AM Pyrimethamine-sulfadoxine resistance in *Plasmodium falciparum*: what next? Trends Parasitol 2001 17 582 588 10.1016/S1471-4922(01)02085-2 11756042

[B19] Zhang Y Meshnick SR Inhibition of *Plasmodium falciparum* dihydropteroate synthetase and growth *in vitro* by sulfa drugs Antimicrob Agents Chemother 1991 35 267 271 10.1128/AAC.35.2.267 2024960 PMC244989

[B20] Basco LK Ringwald P Molecular epidemiology of malaria in Yaoundé, Cameroon. III. Analysis of chloroquine resistance and point mutations in the multidrug resistance 1 (*pfmdr 1*) gene of *Plasmodium falciparum* Am J Trop Med Hyg 1998 59 577 581 9790433 10.4269/ajtmh.1998.59.577

[B21] Duraisingh MT Drakeley CJ Muller O Bailey R Snounou G Targett GA Greenwood BM Warhurst DC Evidence for selection for the tyrosine-86 allele of the *pfmdr 1* gene of *Plasmodium falciparum* by chloroquine and amodiaquine Parasitology 1997 114 205 211 10.1017/S0031182096008487 9075340

[B22] Foote SJ Kyle DE Martin RK Oduola AM Forsyth K Kemp DJ Cowman AF Several alleles of the multidrug-resistance gene are closely linked to chloroquine resistance Nature 1990 345 255 258 10.1038/345255a0 2185424

[B23] Grobusch MP Adagu IS Kremsner PG Warhurst DC *Plasmodium falciparum*: *in vitro* chloroquine susceptibility and allele-specific PCR detection of *Pfmdr1* Asn86Tyr polymorphism in Lambarene, Gabon Parasitology 1998 116 211 217 10.1017/S0031182097002266 9550213

[B24] Basco LK de Pecoulas PE Le Bras J Wilson EN *Plasmodim falciparum*: Molecular characterization of multi drug-resistant Cambodian isolates Exp Parasitol 1996 82 97 103 8617353 10.1006/expr.1996.0013

[B25] Marfurt J Muller I Sie A Oa O Reeder JC Smith TA Beck HP Genton B The usefulness of twenty-four molecular markers in predicting treatment outcome with combination therapy of amodiaquine plus sulphadoxine-pyrimethamine against falciparum malaria in Papua New Guinea Malar J 2008 7 61 10.1186/1475-2875-7-61 18423045 PMC2377269

[B26] Ruetz S Delling U Brault M Schurr E Gros P The *pfmdr1* gene of *Plasmodium falciparum* confers cellular resistance to antimalarial drugs in yeast cells Proc Natl Acad Sci USA 1996 93 9942 9947 10.1073/pnas.93.18.9942 8790436 PMC38534

[B27] Reed MB Saliba KJ Caruana SR Kirk K Cowman AF Pgh1 modulates sensitivity and resistance to multiple antimalarials in *Plasmodium falciparum* Nature 2000 403 906 909 10.1038/35002615 10706290

[B28] Pillai DR Hijar G Montoya I Marquino W Ruebush TK Wrongsrichanalai C Kain KC Lack of prediction of mefloquine and mefloquine-artesunate treatment outcome by mutations in the *Plasmodium falciparum* multidrug resistance 1 (*pfmdr1*) gene for *P. falciparum* malaria in Peru Am J Trop Med Hyg 2003 68 107 110 12557835

[B29] Duraisingh MT Jones P Sambou I von Seidlein L Pinder M Warhurst DC The tyrosine-86 allele of the *pfmdr1* gene of *Plasmodium falciparum* is associated with increased sensitivity to the anti-malarials mefloquine and artemisinin Mol Biochem Parasitol 2000 108 13 23 10.1016/S0166-6851(00)00201-2 10802315

[B30] Duraisingh MT Roper C Walliker D Warhurst DC Increased sensitivity to the antimalarials mefloquine and artemisinin is conferred by mutations in the *pfmdr1* gene of *Plasmodium falciparum* Mol Microbiol 2000 36 955 961 10.1046/j.1365-2958.2000.01914.x 10844681

[B31] Lambros C Vanderberg JP Synchronization of *Plasmodium falciparum* erythrocytic stages in culture J Parasitol 1979 65 418 420 10.2307/3280287 383936

[B32] Bogreau H Renaud F Bouchiba H Durand P Assi SB Henry MC Garnotel E Pradines B Fusai T Wade B Adehossi E Parola P Kamil MO Puijalon O Rogier C Genetic diversity and structure of African *Plasmodium falciparum* populations in urban and rural areas Am J Trop Med Hyg 2006 74 953 959 16760503

[B33] Henry M Diallo I Bordes J Ka S Pradines B Diatta B M’Baye PS Sane M Thiam M Gueye PM Wade B Touze JE Debonne JM Rogier C Fusai T Urban malaria in Dakar, Senegal: chemosusceptibility and genetic diversity of *Plasmodium falciparum* isolates Am J Trop Med Hyg 2006 75 146 151 16837722

[B34] Pascual A Basco LK Baret E Amalvict R Travers D Rogier C Pradines B Use of the atmospheric generators for capnophilic bacteria Genbag CO_2_® for the evaluation of *in vitro Plasmodium falciparum* susceptibility to standard anti-malarial drugs Malar J 2011 10 8 10.1186/1475-2875-10-8 21235757 PMC3031278

[B35] Kaddouri H Nakache S Houzé S Mentré F Le Bras J Assessment of the drug susceptibility of *Plasmodium falciparum* clinical isolates from Africa using a *Plasmodium* lactate dehydrogenase immunodetection assay and an inhibitory maximum effect model for precise measurement of the 50-percent inhibitory concentration Antimicrob Agents Chemother 2006 50 3343 3349 10.1128/AAC.00367-06 17005815 PMC1610081

[B36] Le Nagard H Vincent C Mentré F Le Bras J Online analysis of *in vitro* resistance to antimalarial drugs through nonlinear regression Comput Methods Programs Biomed 2011 104 10 18 10.1016/j.cmpb.2010.08.003 20828858

[B37] Tinto H Ouédraogo JB Erhart A van Overmeir C Dujardin JC van Marck E Guiguemdé TR D’Alessandro U Relationship between the *Pfcrt* T76 and the *Pfmdr-1* Y86 mutations in *Plasmodium falciparum* and *in vitro*/*in vivo* chloroquine resistance in Burkina Faso, West Africa Infect Genet Evol 2003 3 287 292 10.1016/j.meegid.2003.08.002 14636690

[B38] Basco LK Ringwald P Molecular epidemiology of malaria in Cameroon.X. Evaluation of *Pfmdr1* mutations as genetic markers for resistance to amino alcohols and artemisinin derivatives. *Am J* Trop Med Hyg 2002 66 667 671 10.4269/ajtmh.2002.66.667 12224572

[B39] Parola P Pradines B Simon F Carlotti MP Minodier P Ranjeva MP Badiaga S Bertaux L Delmont J Morillon M Silai R Brouqui P Parzy D Antimalarial drug susceptibility and point mutations associated with resistance in 248 *Plasmodium falciparum* isolates imported from Comoros to Marseille Am J Trop Med Hyg 2007 77 431 437 17827355

[B40] Fall B Diawara S Sow K Baret E Diatta B Fall KB Mbate PS Fall F Diémé Y Rogier C Wade B Bercion R Pradines B *Ex vivo* susceptibility of *Plasmodium isolates* from Dakar, Senegal, to seven standard anti-malarial drugs Malar J 2011 10 310 10.1186/1475-2875-10-310 22014157 PMC3210113

[B41] Jambou R Legrand E Niang M Khim N Lim P Volney B Ekala MT Bouchier C Esterre P Fandeur T Mercereau-Puijalon O Resistance of *Plasmodium falciparum* field isolates to *in vitro* artemether and point mutations of the SERCA-type PfATPase6 Lancet 2005 366 1960 1963 10.1016/S0140-6736(05)67787-2 16325698

[B42] Pradines B Rogier C Fusai T Tall A Doury JC Sensibilité *in vitro* de 85 isolats de *Plasmodium falciparum* au Sénégal Med Trop 1996 56 141 145 8926873

[B43] Pradines B Tall A Rogier C Spiegel A Mosnier J Marrama L Fusai T Millet P Panconi E Trape JF Parzy D *In vitro* activities of ferrochloroquine against 55 Senegalese isolates of *Plasmodium falciparum* in comparison with those of standard antimalarial drugs Trop Med Int Health 2002 7 265 270 10.1046/j.1365-3156.2002.00848.x 11903989

[B44] Pradines B Mabika Mamfoumbi M Tall A Sokhna C Koeck JL Fusai T Mosnier J Czarnecki E Spiegel A Trape JF Kombila M Rogier C *In vitro* activity of tafenoquine against the asexual blood stages of *Plasmodium falciparum* isolates from Gabon, Senegal, and Djibouti Antimicrob Agents Chemother 2006 50 3225 3226 10.1128/AAC.00777-06 16940138 PMC1563556

[B45] Gari-Toussaint M Pradines B Mondain V Keundjian A Dellamonica P Le Fichoux Y Sénégal et paludisme. Echec prophylactique vrai de la méfloquine Presse Med 2002 31 1136 12162100

[B46] Hatin I Trape JF Legros F Bauchet J Le Bras J Susceptibility of *Plasmodium falciparum* strains to mefloquine in an urban area in Senegal Bull World Health Organ 1992 70 363 367 1638665 PMC2393281

[B47] Sokhna C Molez JF Ndiaye P Sane B Trape JF Tests *in vivo* de chimiosensibilite de *Plasmodium falciparum* à la chloroquine au Sénégal: évolution de la résistance et estimation de l’efficacité thérapeutique Bull Soc Pathol Exot 1997 90 83 89 9289259

[B48] Pradines B Rogier C Fusai T Tall A Trape JF Doury JC *In vitro* activity of artemether against African isolates (Senegal) of *Plasmodium falciparum* in comparison with standard antimalarial drugs Am J Trop Med Hyg 1998 58 354 357 9546418 10.4269/ajtmh.1998.58.354

[B49] Pradines B Tall A Parzy D Spiegel A Fusai T Hienne R Trape JF Doury JC *In vitro* activity of pyronaridine and amodiaquine against African isolates (Senegal) of *Plasmodium falciparum* in comparison with standard antimalarial agents J Antimicrob Chemother 1998 42 333 339 10.1093/jac/42.3.333 9786473

[B50] Pradines B Tall A Ramiandrasoa F Spiegel A Sokhna C Fusai T Mosnier J Daries W Trape JF Kunesch G Parzy D Rogier C *In vitro* activity of iron-binding compounds against Senegalese isolates of *Plasmodium falciparum* J Antimicrob Chemother 2006 57 1093 1099 10.1093/jac/dkl117 16595639

[B51] Ndiaye D Patel V Demas A LeRoux M Ndir O Mboup S Clardy J Lakshmanan V Daily JP Wirth DF Short report: an non-radioactive DAPI-based high-throughput *in vitro* assay to assess *Plasmodium falciparum* responsiveness to antimalarials – Increased sensitivity of *P. falciparum* to chloroquine in Senegal Am J Trop Med Hyg 2010 82 228 230 10.4269/ajtmh.2010.09-0470 20133997 PMC2813162

[B52] Djimdé A Doumbo OK Cortese JF Kayentao K Doumbo S Diourté Y Dicko A Su XZ Nomura T Fidock DA Wellems TE Plowe CV A molecular marker for chloroquine-resistant *falciparum* malaria N Engl J Med 2001 344 257 263 10.1056/NEJM200101253440403 11172152

[B53] Niang M Marrama L Ekala MT Gaye A Tall A Ndiaye JL Sarr D Dangou JM Lehesran JY Bouchier C Mercereau-Puijalon O Jambou R Accumulation of CIEVT *Pfcrt* allele of *Plasmodium falciparum* in placenta of pregnant women living in an urban area of Dakar, Senegal J Antimicrob Chemother 2008 62 921 928 10.1093/jac/dkn299 18682531

[B54] Thomas SM Ndir O Dieng T Mboup S Wypij D Maguire JH Wirth DF *In vitro* chloroquine susceptibility and PCR analysis of *pfcrt* and *pfmdr1* polymorphisms in *Plasmodium falciparum* isolates from Senegal Am J Trop Med Hyg 2002 66 474 480 12201579 10.4269/ajtmh.2002.66.474

[B55] Sarr O Myrick A Daily J Diop BM Dieng T Ndir O Salif Sow P Mboup S Wirth DF *In vivo* and *in vitro* analysis of chloroquine resistance in *Plasmodium falciparum* isolates from Senegal Parasitol Res 2005 97 136 140 10.1007/s00436-005-1406-7 15986248 PMC2579896

[B56] Sarr O Ahouidi AD Ly O Daily JP Ndiaye D Ndir O Mboup S Wirth DF Mutations in PfCRT K76T do no correlate with sulfadoxine-pyrimethamine-amodiaquine failure in Pikine Parasitol Res 2008 103 765 769 10.1007/s00436-008-1038-9 18523801 PMC2583234

[B57] Bertin G Ndam NT Jafari-Guemouri S Fievet N Renart E Sow S Le Hesran JY Deloron P High prevalence of *Plasmodium falciparum pfcrt* K76T mutation in pregnant women taking chloroquine prophylaxis in Senegal J Antimicrob Chemother 2005 55 788 791 10.1093/jac/dki097 15814601

[B58] Wurtz N Fall B Pascual A Diawara S Sow K Baret E Diatta B Fall KB Mbaye PS Fall F Diémé Y Rogier C Bercion R Briolant S Wade B Pradines B Prevalence of molecular markers of *Plasmodium falciparum* drug resistance in Dakar Senegal. Malar J 2012 11 197 10.1186/1475-2875-11-197 PMC3470961 22694921

[B59] Ndiaye M Faye B Tine R Ndiaye JL Lo A Abiola A Dieng Y Ndiaye D Hallett R Alifrangis M Gaye O Assessment of the molecular marker of *Plasmodium falciparum* chloroquine resistance (*Pfcrt*) in Senegal after several years of chloroquine withdrawal Am J Trop Med Hyg 2012 87 640 645 10.4269/ajtmh.2012.11-0709 22927495 PMC3516312

[B60] Ly O Gueye PE Deme AB Dieng T Badiane AS Ahouidi AD Diallo M Bei AK Wirth DF Mboup S Sarr O Evolution of the *pfcrt* T76 and *pfmdr1* Y86 markers and chloroquine susceptibility 8 years after cessation of chloroquine use in Pikine, Senegal Parasitol Res 2012 111 1541 1546 10.1007/s00436-012-2994-7 22706959

[B61] Gardella F Assi S Simon F Bogreau H Eggelte T Ba F Foumane V Henry MC Traore Kientega P Basco L Trape JF Lalou R Martelloni M Desbordes M Baragatti M Briolant S Almeras L Pradines B Fusai T Rogier C Antimalarial drug use in general populations of tropical Africa Malar J 2008 7 124 10.1186/1475-2875-7-124 18611279 PMC2494551

[B62] Frosch AEP Venkatesan M Laufer MK Patterns of chloroquine use and resistance in sub-Saharan Africa: a systematic review of household survey and molecular data Malar J 2011 10 116 10.1186/1475-2875-10-116 21554692 PMC3112453

[B63] Ministère de la Santé et de la Prévention Médicale Enquête nationale sur le paludisme au Sénégal 2006 2007 http://www.measuredhs.com/pubs/pdf/MIS1/MIS1.pdf

[B64] Ministère de la Santé et de la Prévention Médicale Enquête nationale sur le paludisme au Sénégal 2008–2009 2009 http://www.measuredhs.com/publications/publication-MIS5-MIS-Final-Reports.cfm

[B65] Kublin JG Cortese JF Njunju EM Mukadam RA Wirima JJ Kazembe PN Djimdé AA Kouriba B Taylor TE Plowe CV Remergence of chloroquine-sensitive *Plasmodium falciparum* malaria after cessation of chloroquine in Malawi J Infect Dis 2003 187 1870 1875 10.1086/375419 12792863

[B66] Laufer MK Thesing PC Eddington ND Masonga R Dzinjalamala FK Takala SL Taylor TE Plowe CV Return of chloroquine antimalarial efficacy in Malawi N Engl J Med 2006 355 1959 1966 10.1056/NEJMoa062032 17093247

[B67] Noranate N Durand R Tall A Marrama L Spiegel A Sokhna C Pradines B Cojean S Guillotte M Bischoff E Ekala MT Bouchier C Fandeur T Ariey F Patarapotikul J Le Bras J Trape JF Rogier C Mercereau-Puijalon O Rapid dissemination of *Plasmodium falciparum* drug resistance despite strictly controlled antimalarial use Plos One 2007 1 139 10.1371/journal.pone.0000139 PMC1764034 17206274

[B68] Agnamey P Brasseur P de Pecoulas E Vaillant M Olliaro P ***Plasmodium falciparum in vitro *****susceptibility to antimalarial drugs in Casamance** (**Southwestern Senegal**) **during the first 5 years of routine use of artesunate**-**amodiaquine** Antimicrob Agents Chemother 2006 50 1531 1534 10.1128/AAC.50.4.1531-1534.2006 16569876 PMC1426962

[B69] Holmgren G Gil JP Ferreira PM Veiga MI Obonyo CO Bjorkman A Amodiaquine resistant *Plasmodium falciparum* malaria *in vivo* is associated with selection of *pfcrt* 76T and *pfmdr1* 86Y Infect Gen Evol 2006 6 309 314 10.1016/j.meegid.2005.09.001 16271310

[B70] Nsobya SL Dokomajilar C Joloba M Dorsey G Rosenthal PJ Resistance-mediating *Plasmodium falciparum pfcrt* and *pfmdr1* alleles after treatment with artesunate-amodiaquine in Uganda Antimicrob Agents Chemother 2007 51 3023 3025 10.1128/AAC.00012-07 17562798 PMC1932488

[B71] Echeverry DF Holmgren G Murillo C Higuita JC Gil JP Osorio L Polymorphisms in the *pfcrt* and *pfmdr1* genes of *Plasmodium falciparum* and *in vitro* susceptibility to amodiaquine and desethylamodiaquine Am J Trop Med Hyg 2007 77 1034 1038 18165517

[B72] Danquah I Coulibaly B Meissner P Petruschke I Muller O Mockenhaupt FP Selection of *pfmdr1* and *pfcrt* alleles in amodiaquine treatment failure in north-western Burkina Faso Acta Trop 2010 114 63 33 10.1016/j.actatropica.2009.12.008 20060374

[B73] Brasseur P Guiguemde R Diallo S Guiyedi V Kombila M Ringwald P Olliaro P Amodiaquine remains effective for treating ucomplicated malaria in West and Central Africa Trans R Soc Trop Med Hyg 1999 93 645 650 10.1016/S0035-9203(99)90083-4 10717757

[B74] Ndiaye JL Faye B Diouf AM Kuété T Cisse M Seck PA Brasseur P Same-Ekobo A Lameyre V Gaye O Randomized, comparative study of the efficacy and safety of artesunate plus amodiaquine, administered as a single daily intake versus two daily intakes in the treatment of uncomplicated *falciparum* malaria Malar J 2008 7 16 10.1186/1475-2875-7-16 18205945 PMC2259365

[B75] Ndiaye JL Randrianarivelojosia M Sagara I Brasseur P Ndiaye I Faye B Randrianasolo L Ratsimbasoa A Forlemu D Ama Moor V Traore A Dicko Y Dara N Lameyre V Diallo M Djimdé A Same-Ekobo A Gaye O Randomized, multicentre assessment of the efficacy and safety of ASAQ – a fixed dose artesunate-amodiaquine combination therapy in the treatment of uncomplicated *Plasmodium falciparum* malaria Malar J 2009 8 125 10.1186/1475-2875-8-125 19505304 PMC2698916

[B76] Dondorp AM Nosten F Yi P Das D Phyo AP Tarning J Lwin KM Ariey F Hanpithakpong W Lee SJ Ringwald P Silamut K Imwrong M Chotivanish K Lim P Herdman T An SS Yeung S Singhasivanon P Day NPJ Lindegardh N Socheat D White NJ Artemisinin resistance in *Plasmodium falciparum* malaria N Engl J Med 2009 361 455 467 10.1056/NEJMoa0808859 19641202 PMC3495232

[B77] Noedl H Se Y Schaecher K Smith BL Socheat D Fukuda MM Evidence of artemisinin-resistant malaria in western Cambodia N Engl J Med 2008 359 2619 2620 10.1056/NEJMc0805011 19064625

[B78] Sibley CH Barnes KI Plowe CV The rationale and plan for creating a World Antimalarial Resistance Network (WARN) Malar J 2007 6 118 10.1186/1475-2875-6-118 17822531 PMC2000470

[B79] Sibley CH Barnes KI Watkins WM Plowe CV A network to monitor antimalarial drug resistance: a plan for moving forward Trends Parasitol 2008 24 43 48 10.1016/j.pt.2007.09.008 18042432

[B80] Pradines B Tall A Fusai T Spiegel A Hienne R Rogier C Trape JF Le Bras J Parzy D *In vitro* activities of benflumetol against 158 Senegalese isolates of *Plasmodium falciparum* in comparison with those of standard antimalarial drugs Antimicrob Agents Chemother 1999 43 418 420 9925550 10.1128/aac.43.2.418 PMC89095

[B81] Brandicourt O Druilhe P Diouf F Brasseur P Turk P Danis M Decreased sensitivity to chloroquine and quinine of some *Plasmodium falciparum* strains from Senegal in September 1984 Am J Trop Med Hyg 1986 35 717 721 3524287 10.4269/ajtmh.1986.35.717

[B82] Jelinek T Schelbert P Loscher T Eichenlaub D Quinine resistant *falciparum* malaria acquired in east Africa Trop Med Parasitol 1995 46 38 40 7631126

[B83] Palmieri F Petrosillo N Paglia MG Conte A Goletti D Pucillo LP Menegon M Sannella A Severini C Majori G Genetic confirmation of quinine-resistant *Plasmodium falciparum* malaria followed by postmalaria neurological syndrome in a traveler from Mozambique J Clin Microbiol 2004 42 5424 5426 10.1128/JCM.42.11.5424-5426.2004 15528762 PMC525244

[B84] Pradines B Pistone T Ezzedine K Briolant S Bertaux L Receveur MC Parzy D Millet P Rogier C Malvy D Quinine-resistant malaria in traveler returning from Senegal, 2007 Emerg Infect Dis 2010 16 546 548 10.3201/eid1603.091669 20202443 PMC3322041

[B85] Pradines B Spiegel A Rogier C Tall A Mosnier J Fusai T Trape JF Parzy D Antibiotics for prophylaxis of *Plasmodium falciparum* infections: *in vitro* activity of doxycycline against Senegalese isolates Am J Trop Med Hyg 2000 62 82 85 10761729 10.4269/ajtmh.2000.62.82

[B86] Briolant S Baragatti M Parola P Simon F Tall A Sokhna C Hovette P Mabika Mamfoumbi M Koeck JL Delmont J Spiegel A Castello J Gardair JP Trape JF Kombila M Minodier P Fusai T Rogier C Pradines B Multinormal *in vitro* distribution model suitable for the distribution of *Plasmodium falciparum* chemosusceptibility to doxycycline Antimicrob Agents Chemother 2009 53 688 695 10.1128/AAC.00546-08 19047651 PMC2630600

[B87] Pradines B Rogier C Fusai T Mosnier J Daries W Baret E Parzy D *In vitro* activities of antibiotics against *Plasmodium falciparum* are inhibited by iron Antimicrob Agents Chemother 2001 45 1746 1750 10.1128/AAC.45.6.1746-1750.2001 11353621 PMC90541

[B88] Ndiaye D Dailly JP Sarr O Ndir O Gaye O Mboup S Wirth DF Mutations in *Plasmodium falciparum* dihydrofolate reductase and dihydropteroate synthase genes in Senegal Trop Med Int Health 2005 10 1176 1179 10.1111/j.1365-3156.2005.01506.x 16262743 PMC2582373

[B89] Cissé B Cairns M Faye E NDiaye O Faye B Cames C Cheng Y NDiaye M Colle Lo A Simondon K Trape JF Faye O NDiaye JL Gaye O Greenwood B Milligan P Randomized trial of piperaquine with sulfadoxine-pyrimethamine or dihydroartemisinin for malaria intermittent preventive treatment in children Plos One 2009 4 7164 10.1371/journal.pone.0007164 PMC2747010 19784374

[B90] Faye B Ndiaye M Ndiaye JL Annie A Tine RC Collé Lo A Ndiaye M Sow D De Sousa A Gaye O Prevalence of molecular markers of *Plasmodium falciparum* resistance to sulfadoxine-pyrimethamine during the intermittent preventive treatment in infants coupled with expanded program immunization in Senegal Parasitol Res 2011 109 133 138 10.1007/s00436-010-2236-9 21207062

[B91] Sokhna C Cissé B Ba EH Milligan P Hallett R Sutherland C Gaye O Boulanger D Simondon K Simondon F Targett G Lines J Greenwood B Trape JF A trial of the efficacy, safety and impact on drug resistance of four drug regimens for seasonal intermittent preventive treatment for malaria in Senegalese children Plos One 2008 3 1471 10.1371/journal.pone.0001471 PMC2198946 18213379

[B92] Cissé B Sokhna C Boulanger D Milet J Ba EH Richardson K Hallett R Sutherland C Simondon K Simondon F Alexander N Gaye O Targett G Lines J Greenwood B Trappe JF Seasonal intermittent preventive treatment with artesunate and sulfadoxine-pyrimethamine for prevention of malaria in Senegalese children: a randomized, placebo-controlled, double-blind trial Lancet 2006 367 659 667 10.1016/S0140-6736(06)68264-0 16503464

